# Dengue Virus 2 American-Asian Genotype Identified during the 2006/2007 Outbreak in Piauí, Brazil Reveals a Caribbean Route of Introduction and Dissemination of Dengue Virus in Brazil

**DOI:** 10.1371/journal.pone.0104516

**Published:** 2014-08-15

**Authors:** Leandra Barcelos Figueiredo, Tetsu Sakamoto, Luiz Felipe Leomil Coelho, Eliseu Soares de Oliveira Rocha, Marcela Menezes Gomes Cota, Gustavo Portela Ferreira, Jaquelline Germano de Oliveira, Erna Geessien Kroon

**Affiliations:** 1 Departamento de Microbiologia, Universidade Federal de Minas Gerais, Belo Horizonte, Minas Gerais, Brazil; 2 Laboratório de Biodados, Universidade Federal de Minas Gerais, Belo Horizonte, Minas Gerais, Brazil; 3 Laboratório de Vacinas, Universidade Federal de Alfenas, Alfenas, Minas Gerais, Brazil; 4 Laboratório de Vírus, Universidade Federal de Minas Gerais, Belo Horizonte, Minas Gerais, Brazil; 5 Laboratório de Biotecnologia, Universidade Federal do Piauí, Parnaíba, Piauí, Brazil; 6 Laboratório de Imunologia Celular e Molecular, Fundação Oswaldo Cruz, Belo Horizonte, Minas Gerais, Brazil; 7 Laboratório de Vírus, Departamento de Microbiologia, Instituto de Ciências Biológicas, Universidade Federal de Minas Gerais, Belo Horizonte, Minas Gerais, Brazil; Singapore Immunology Network, Agency for Science, Technology and Research (A*STAR), Singapore

## Abstract

*Dengue virus* (DENV) is the most widespread arthropod-borne virus, and the number and severity of outbreaks has increased worldwide in recent decades. Dengue is caused by DENV-1, DENV- 2, DENV-3 and DENV-4 which are genetically distant. The species has been subdivided into genotypes based on phylogenetic studies. DENV-2, which was isolated from dengue fever patients during an outbreak in Piaui, Brazil in 2006/2007 was analyzed by sequencing the envelope (E) gene. The results indicated a high similarity among the isolated viruses, as well as to other DENV-2 from Brazil, Central America and South America. A phylogenetic and phylogeographic analysis based on DENV-2E gene sequences revealed that these viruses are grouped together with viruses of the American-Asian genotype in two distinct lineages. Our results demonstrate the co-circulation of two American-Asian genotype lineages in northeast Brazil. Moreover, we reveal that DENV-2 lineage 2 was detected in Piauí before it disseminated to other Brazilian states and South American countries, indicating the existence of a new dissemination route that has not been previously described.

## Introduction

Dengue is the most significant mosquito-borne viral disease that affects humans. Of all members of the *Flavivirus* genus, the *Dengue virus* (DENV) is responsible for the highest morbidity and mortality rates. DENV infection is endemic in more than 100 countries, with tens of millions of cases of dengue fever (DF) recorded per year, including up to 500,000 cases of dengue hemorrhagic fever/dengue shock syndrome (DHF/DSS), which require hospitalization for supportive treatment [1]–[2].

DENV is most commonly transmitted by the mosquito vector *Aedes aegypti*; however, it can also be transmitted by other members of the genus *Aedes*, including *Aedes albopictus*. The majority of infected humans is asymptomatic or develops DF, an acute febrile illness [3]–[5].

DENV belongs to the *Flavivirus* genus of the *Flaviviridae* family and has four genetically and antigenically distinct serotypes: DENV-1, DENV-2, DENV-3 and DENV-4. The virus is enveloped with a single-stranded, positive-sense RNA genome of approximately 11 kb containing a single open reading frame flanked by untranslated regions (5' and 3' UTRs) [6]–[7].

Phylogenetic and molecular analyses based on nucleic acid sequence data have been used to analyze the genetic variation of DENV, to characterize DENV serotypes and for epidemiological studies [8]–[11]. These approaches have revealed extensive variability among the DENVs, leading to the recognition of different genotypes within each species. Five DENV-2 genotypes have been described: Asian I (AS-I), Asian II (AS-II), American-Asian (AM/AS), Cosmopolitan (COS) and American (AM) [8], [12]–[14]. In Brazil, DENV-2 was first identified in the state of Rio de Janeiro in 1990, and its introduction in Brazil resulted in several DF cases and the first severe forms of DHF as well as fatal cases of DSS [15]–[17]. This event was followed by a rapid spread of DENV-2 to other Brazilian states. [18]–[19]. An analysis of the envelope (E) gene of isolates from Rio de Janeiro and São Paulo collected during 2007–2008 and 2010 revealed that at least two lineages of the American-Asian genotype of DENV-2 have circulated in Brazil [20]–[22]. In addition, the virus that circulated in São Paulo in 2010 was closely related to the virus that circulated in Rio de Janeiro in 2007 and 2008 [21].

**Figure 1:**
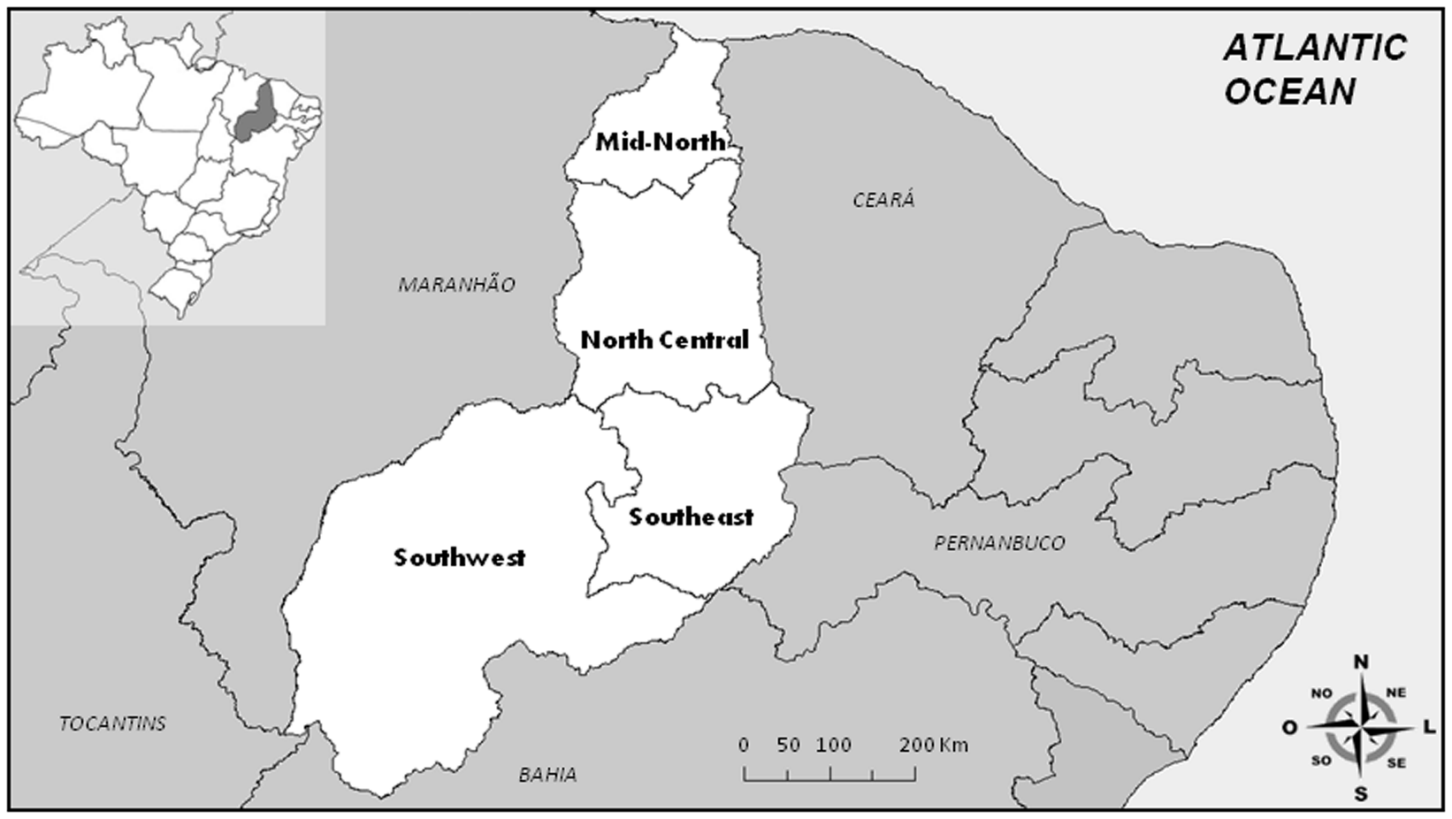
Geographic location of Piauí and its meso-regions. The state of Piauí is located in northeastern Brazil and is divided into the Mid-North, North Central, Southeast and Southwest regions. The state capital, Teresina, is indicated with an arrow on the map.

Piauí is located in the northeast region of Brazil; it has an area of 252,378 km^2^ and a population of 3,118,360 (IBGE, 2010 [23]), and its northern region borders the Atlantic Ocean (Figure 1). It also borders the states of Maranhão (W), Ceará and Pernambuco (E) and Bahia and Tocantins (SW) [24]. According to the Ministry of Health, the first cases of dengue in the state of Piauí were reported in 1995, and in 2006, this state reported 4,759 cases of dengue. During 2006–2007, virological surveillance revealed that DENV-2 and DENV-3 were the most prevalent viruses in Piauí (data not published).

In the present study, we focused on the molecular epidemiology of DENV-2 isolates with different clinical manifestations from the outbreak in Piauí in 2006/2007 using Bayesian phylogeographic methods. Our phylogenetic analyses demonstrated the co-circulation of two different lineages of the DENV-2 American-Asian genotype in the state of Piauí during this outbreak, making this study the first report of co-circulation of different DENV-2 lineages in the same outbreak. Furthermore, by applying a spatiotemporal dynamics analysis, we suggest a new route of introduction of DENV-2 in Brazil.

## Methods

### Ethics Statement

This study was approved by the Committee of Ethics in Research of the Universidade Federal de Minas Gerais (number 415/04) and the blood collected was approved to be used in the research as unlinked anonymous samples.

### Epidemiological Study and Clinical Samples

To study the epidemiological profile of dengue, 4,564 serum samples were collected from patients suspected of having febrile dengue who reported to health community centers distributed throughout the state of Piauí (northeast Brazil) during 2006/2007. Serum was collected six days after symptom onset and sent to the Central Laboratory to confirm DENV infection via detection of specific IgM. Dengue cases were classified according to criteria established by the World Health Organization [1]. Demographic data (sex, age and location) were obtained from the standard notification form for dengue. Abiotic factors (pluviosity and average temperature) were obtained from the Agrometeorology Monitoring System of the Food and Animal Ministry (http://www.agritempo.gov.br).

Serum samples were obtained from a select subgroup of 46 patients in the acute phase without any type of hemorrhagic manifestation. The samples were collected six days after the osymptom onset, and stored at −70°C until use in viral isolation procedures and reverse transcription polymerase chain reactions (RT-PCR).

### Detection of dengue IgM antibodies by ELISA

All sera were tested using the Dengue IgM Capture ELISA kit (Panbio, Australia) following the manufacturer's instructions.

### Viral isolation in cell cultures

*Aedes albopictus*C6/36 cells were propagated in LeibovitzL15 medium (Gibco, USA) supplemented with 10% fetal calf serum (Cultilab, Brazil) at 28°C [25]–[26]. For virus isolation, a 50 µl serum sample was incubated with C6/36 cells supplemented with 2% fetal calf serum. Each sample was passaged at least three times. Infected C6/36 cells showing typical cytopathic effects were harvested, and the cell culture supernatants were used for viral RNA extraction. Uninfected cells used as controls were treated similarly, although the serum was omitted and replaced by medium. The cells inoculated with serum samples that did not show cytopathic effects even after the fifth passage were considered negative.

### RNA extraction and RT-PCR

The QIAampViral RNA kit (Qiagen, USA) was used to extract viral RNA from the cell culture supernatants. Extracted RNA was stored at −70°C or immediately subjected to (RT-PCR. Virus in the cell cultures was directly detected and identified by RT-PCR according to a method developed by Lanciotti and colleagues [27] using primers specific for the C-prM gene. Target viral RNA was converted to cDNA using Moloney murine leukemia virus (M-MLV) reverse transcriptase (Promega, Corp., Madison, WI) and the consensus downstream primer D2. The cDNA was then amplified by PCR using *Taq* polymerase (Promega, Corp., Madison, WI) and consensus D1 and D2 primers. For the sequencing of isolates determined to be positive for DENV-2, specific primers for the E gene DNA that are able to detect all DENV-2 isolates were used. The primers designed to amplify a 945 bp fragment and a 651 bp fragment were D2 1EF (5'-GCTGTCGCTCCTTCAATG-3′), D2 2ER (5′-TTC TGC TAT TTC CTT CAC-3′), D2 3EF (5'-GTTCACGGGACATCTCAA -3)' and D2 4ER (5'-GTTCTTTATTTTTCCAGC- 3'). The amplification was carried out in a thermal cycler (Eppendorf Mastercycler-gradient 96-well,USA) as follows: 30 cycles at 95°C for 45 seconds, 60°C for 45 seconds, and 72°C for 120 seconds, followed by a final incubation at 72°C for 10 minutes. The PCR products were fractionated by agarose gel electrophoresis, stained with SYBR Safe DNA gel stain (Invitrogen, USA) and visualized under an UV transilluminator.

### Nucleotide sequencing

The nucleotide sequence of the E gene was determined in eight isolates that were identified as DENV-2 by RT-PCR. The PCR-amplified E gene DNA was purified (QIAquick PCR purification kit-Qiagen, USA) and used directly in sequencing reactions. Each DNA sample was sequenced at least five times in both orientations (MegaBACE sequencer, GE Healthcare, USA). All sequences generated in this work were deposited in GenBank (http://www.ncbi.nlm.nih.gov), and their respective accession numbers are provided in Table 1.

**Table 1:** Description of DENV-2 isolates from Piauí, Brazil.

Isolate	Disease	Sex^a^	AGE (YEARS)	Collection day	Passage b history	GenBank number
PI-55	FD	M	29	2	5^th^	KJ147102
PI-58	FD	M	40	-	5^th^	KJ147098
PI-59	FD	M	17	3	4^th^	KJ147100
PI-62	FD	F	30	3	4^th^	KJ147099
PI-74	FD	M	-	-	3^rd^	KJ147097
PI-82	FD	F	8	6	3^rd^	KJ147095
PI-83	FD	F	21	4	3^rd^	KJ147101
PI-111	FD	F	41	2	4^th^	KJ147096

a F is female and M is male.

b Number of passages in cell culture (C6/36 cell line) are shown.

### Evolutionary analyses

To determine the evolutionary relationship of DENV-2 isolated from the state of Piauí with other isolates described in previous studies, we performed an analysis based on the Maximum Likelihood (ML) and Bayesian Inference (BI) methods.

DENV-2 E gene sequences deposited in the GenBank database were retrieved after a sequence similarity search using the BLAST algorithm [28]; the E genes obtained in this work were used as a query. A total of 43 samples were manually selected for further evolutionary analysis. All selected samples belong to the genotype American/Asian. From the same database, we also retrieved the E gene sequences of isolates representative of all five DENV-2 genotypes: American (3 sequences), Asian II (3 sequences), Asian I (5 sequences) and Cosmopolitan (10 sequences). All sequences used in this work are listed in Table S1 and are presented in the following format in all phylogenetic trees: gi number/country/year of isolation. Samples from Brazil are identified as follows: gi number/city-state-BR/year of isolation. Retrieved nucleotide sequences, totalizing 64 sequences, were aligned with 8 DENV-2 E gene sequences from the state of Piauí using the software MUSCLE [29]. Selection of the best-fit nucleotide substitution model was performed using jModel Test [30]. Based on the Bayesian Information Criterion (BIC), the Tamura-Nei model with gamma correction (TN93+G) was the best-fit model and was used for phylogenetic tree reconstruction using ML and BI methods. Phylogenetic tree reconstruction using ML methods was performed using PhyML v.3.1 [31] with 100 bootstrap replicates. The final tree was then visualized in Fig Tree v. 1.4.0 [32].

To infer the time to the most recent common ancestor (TMRCA) and to trace the geographic flow of DENV-2 over time, we used the Markov Chain Monte Carlo algorithm implemented in the BEAST package [33]–[34].The *clocklikeness* of our DENV-2 datasets was examined using PATH-O-GEN software v.1.4 [35] using the ML tree generated by PhyMLas input [31]. This analysis revealed a high correlation coefficient (0.88) and R squared value (0.77), indicating that the data fit to a strict molecular clock model. The estimated mutation rate (slope rate) was 7.55E-4 substitution/site/year, which is in the range of the mutation rate for E genes of DENV-2 described in previous studies [36]–[37].

The date of isolation of each sample was used as a calibration point to estimate the divergence time in years. In addition, information about the specific location of each sample (Table S1) was assigned to each sequence, and a discrete phylogeographic analysis was performed. Runs were conducted using an extended Bayesian Skyline Plot coalescent model for 400,000,000 generations with sampling every 10,000 generations. The effective sample sizes (ESS) for each parameter sampled were examined using TRACER v.1.5 [38]. Posterior trees were summarized by discarding the first 10% of the sampled trees and choosing the Maximum Clade Credibility (MCC) tree from the remaining trees using TREEANNOTATOR v.1.7.5 [39]. The final tree was then visualized in Fig Tree v. 1.4.0 [32].

## Results

### Epidemiological and laboratory findings showed a high number of serum samples that were positive for dengue virus by ELISA

Piauí is located in the northeast region of Brazil, and it is considered an area of high endemicity (Figure 1). A total of 4,564 samples were analyzed by ELISA: 1,742 (38.2%) were DENV IgM-positive and 2,822 (61.8%) were DENV IgM-negative. The highest number of suspected and ELISA positive serum samples were obtained in May and June, which is a period that was preceded by intense precipitation (Figure S1).The majority of IgM-positive dengue cases were women (1,080/62%). The most highly affected age group were individuals who were 16–30 years old, accounting for 29.4% (512 cases) of the IgM-positive samples, followed by those who were 31–45 years old, accounting for 26.2% (457 cases) of the IgM-positive samples (Table 2).

**Table 2:** Age and sex distribution of IgM-ELISA dengue positive patients from the 2006/2007 outbreak in Piauí, Brazil.

Age	Male		Female		Total	
	Number	%	Number	%	Number	%
0 a≤5	34	5,1	29	2,7	63	3,6
5 a15	105	15,9	116	10,7	221	12,7
16 a 30	192	29,0	320	29,6	512	29,4
31 a 45	155	23,4	302	28,0	457	26,2
46 a 60	83	12,5	192	17,8	275	15,8
>60	64	9,7	85	7,9	149	8,6
NI	29	4,4	36	3,3	65	3,7
Total	662	100	1080	100	1742	100

NI – not informed.

DENV-2 was isolated from eight of 46 acute-phase serum samples tested. The demographic and clinical information for these patients is summarized in Table 1. Microscopic examination of cells inoculated with serum from the patients showed a clearly visible cytopathic effect with changes in the monolayer, such as syncytial cell formation and cytoplasmic vacuoles, after the third passage.

### Isolates from Piauí show a high level of identity with the American-Asian genotype of DENV-2

Pairwise alignment of the DENV-2 E gene sequences obtained in this work (1,344 nucleotides out of 1,485 nucleotides) showed percentages identities ranging from 96.2% to 99.9% for nucleotides (nt) and 98.4% to 100% for amino acids (aa) (Table S2). Lower identity values were found when the sequences were compared with the isolate PI-111 (KJ147096). Although the sample PI-111 is more distantly related to other samples from Piauí, when all these sequences were aligned and compared with several DENV-2 E gene sequences isolated worldwide (Table S2), all the samples showed a higher level of identity (96.06–99.93%) with isolates belonging to the American-Asian genotype. The genotype classification of samples from Piauí as American-Asian was further confirmed by phylogenetic analysis.

### The dengue outbreak in the state of Piauí (2006/2007) involved the co-circulation of two DENV-2 lineages

**Figure 2:**
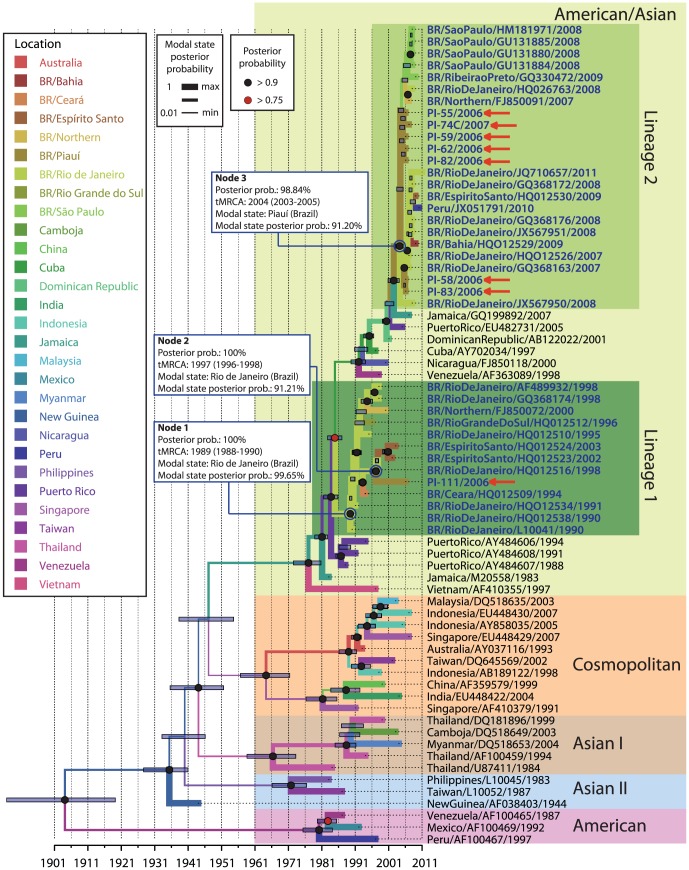
Evolutionary relationship between DENV-2 isolates from Piauí and the five genotypes of DENV-2. A maximum clade credibility (MCC) tree was selected after Bayesian inference analysis (strict molecular clock; TN93+G; 400,000,000 iterations) of 72 DENV-2 envelope sequences. The five DENV-2 genotypes (American-Asian, American, Asian I, Asian II and Cosmopolitan) are highlighted in different colors. Brazilian isolates (bold blue letters) clustered within the American-Asian genotype and could be divided into two groups: lineage 1(dark green block) and lineage 2 (light green block). Each node is represented by colours black and red (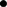), which presented posterior probability value>0.9 and >0.75, respectively. Blue bars represent the extent of the 95% highest probability density (95% HPD) for each divergence time. The most probable geographic state for each internal node was inferred by discrete phylogeographic analysis. Different colors in the branch represent distinct geographical states according to the legend on the left side of the figure. Branch width is proportional to the probability value of the inferred ancestral geographical state.

A consensus ML tree and BI tree were built using the nucleotide sequences of DENV-2 E genes obtained in this work and those retrieved from the GenBank database (listed in Table S1). In this analysis, all five genotypes (American-Asian, American, Asian I, Asian II and Cosmopolitan) are represented. Both the ML tree (Figure S2) and BI tree (Figure 2) show that all sequenced samples from Piauí during the 2006/2007 outbreak clustered with the American-Asian genotype and other Brazilian DENV-2 isolates. However, samples from Piauí were separated in two different lineages, referred to as lineage 1 and lineage 2, with high posterior probability support in the branch (Figure 2). Lineage 1 is characterized to include older DENV2 isolates from various states of Brazil (1990–2003), whereas lineage 2 includes more recent Brazilian DENV-2 samples (2007–2010). Among samples from Piauí, one sample, PI-111 (Piauí/Brazil/2006) grouped in lineage 1, and the other seven isolates, PI-55, PI-58, PI-59, PI-62,PI-82, PI-83 (Piauí/Brazil/2006) and PI-74 (Piauí/Brazil/2007) clustered in lineage 2. The detection of samples from Piauí in both lineages is an evidence of a temporal co-circulation of two genetically distinct DENV-2 lineages in this state.

**Figure 3:**
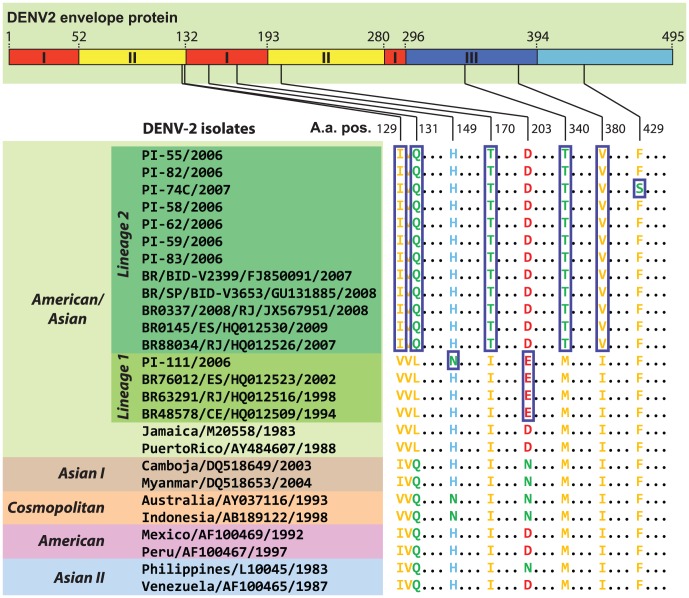
Amino acid polymorphisms in the envelope protein of the American/Asian DENV-2 genotype. (Top) A diagram of the DENV-2 envelope protein showing its three protein domains (I, II and III). (Bottom) A partial alignment of the envelope protein showing sites of amino acid polymorphism within the American-Asian genotype. Other DENV-2 genotypes (Asian I, Asian II, Cosmopolitan and American) are shown for comparison. Amino acid sites in blue boxes are those that most likely had underwent a non-synonymous mutation after the divergence of the two lineages because they differ from the two ancient American-Asian DENV-2 samples (Jamaica/M20558/1983 and Puerto Rico/AY484607/1988). Amino acids are colored according to their side chain charge (hydrophobic: yellow; polar: green; negatively charged: red; and positively charged: blue).

By comparing the amino acid sequences from DENV-2 American-Asian genotype lineages 1 and 2 with older DENV-2 samples of the American-Asian genotype in the phylogenetic tree (Jamaica/M20558/1983 and Puerto Rico/AY484607/1988) (Figure 3), we observed that samples from lineage 1 are characterized by one amino acid substitution D203E, whereas samples from lineage 2 have 5 amino acid substitutions: V129I, L131Q, I170T, M340T and I380V. Additionally, the isolate PI-111 from lineage 1, exhibited the H149N substitution, and the same amino acid was encountered in some samples from Asian I and Cosmopolitan genotypes. The F429S amino acid substitution is also observed in PI-74C, an isolate from lineage 2.

### Introduction of the DENV-2 American-Asian genotype lineage 2 in Brazil occurred earlier than previously described

Phylogeographic analysis (Figure 2) from the same sequence dataset used for ML tree reconstruction estimated the time of introduction of both American-Asian DENV-2 lineages in Brazil. For lineage 1, coalescent analysis inferred that the MRCA of Brazilian samples originated from the state of Rio de Janeiro (modal state posterior probability  = 99.65%) and had existed since 1989 (95% HPD  = 1988–1990) (Figure 2, Node 1). Later, the virus spread to other Brazilian states, including Piauí. Introduction of this virus in Piauí was estimated to have occurred in 1997 (95% HPD  = 1996–1998) (Figure 2, Node 2). In lineage 2, all samples from Piauí were positioned at the base of the branch (Figure 2). Coalescent analysis suggests that, in this lineage, the MRCA of Brazilian DENV-2 has existed since 2004 (95% HPD  = 2003–2005) and was inferred to be from Piauí (modal state posterior probability  = 91.20%). This virus most likely originated from a country in Central America, such as Jamaica, and later spread to other Brazilian states and was introduced to other countries in South America, such as Peru.

**Figure 4:**
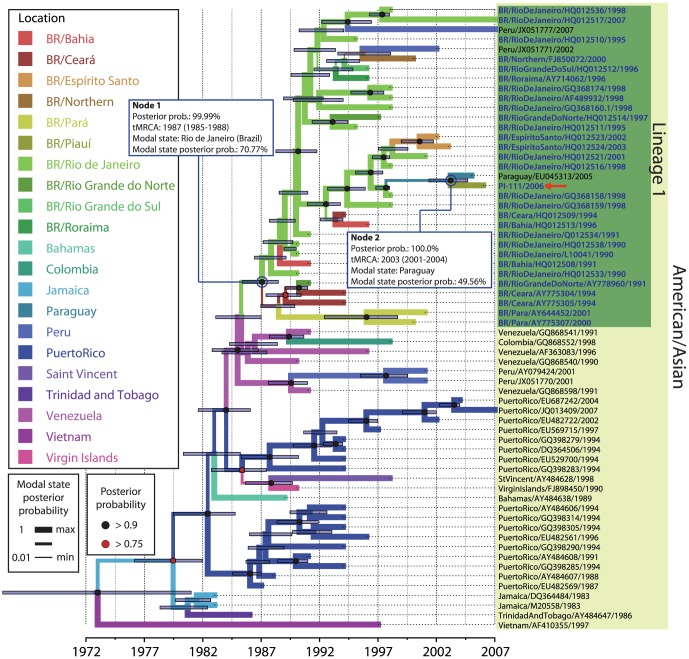
Bayesian coalescent and discrete phylogeographic analyses of Brazilian DENV-2 lineage 1 based on envelope nucleotide sequences. A maximum clade credibility tree was inferred by Bayesian inference analysis (strict molecular clock; TN93+G; 200,000,000 iterations) using 63 DENV-2 envelope sequences (summarized in Table S1) retrieved by a BLAST search against the entire GenBank database and using PI-111/2006 (indicated by a red arrow) as a query.PI-111/2006 is an isolate from the state of Piauí (Brazil) that clustered in Brazilian DENV-2 lineage 1. Nodes that presented posterior probability value of >0.9 and >0.75 are represented by black and red circle (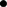), respectively. Blue bars in each node represent the extent of the 95% highest probability density (95% HPD) for each divergence time. The most probable geographic state for each internal node was inferred by discrete phylogeographic analysis. Different colors in the branch represent distinct geographical states according to the legend on the left side of the figure. The branch width is proportional to the probability value of the inferred ancestral geographical state.

**Figure 5:**
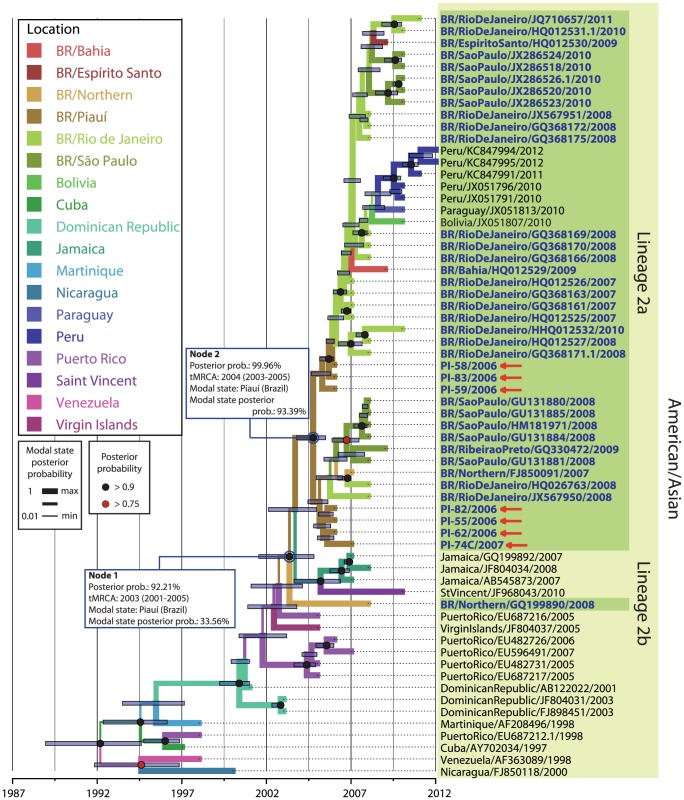
Bayesian coalescent and discrete phylogeographic analyses of Brazilian DENV-2 lineage 2 based on envelope nucleotide sequences. A maximum clade credibility tree was inferred by Bayesian inference analysis (strict molecular clock; TN93+G; 200,000,000 iterations). The 57 DENV-2 envelope sequences (summarized in Table S1) in the tree were retrieved using the BLASTn algorithm; envelope sequences from Piauí isolates (Brazil, indicated by red arrow) clustered in the Brazilian DENV-2 lineage 2 were used as a query. Brazilian isolates (in bold blue letters) weresubdivided into two subgroups that referred as lineage 2a and 2b, respectively. Nodes that presented posterior probability value of >0.9 and >0.75 are represented by black and red circle (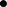), respectively. Different colors in the branch represent the different geographical states according to the legend on the left side of the figure. The branch width is proportional to the probability value of the inferred ancestral geographical state.

To obtain more detail about the evolutionary history of both lineages of DENV-2 that were circulating in Piauí during 2006–2007, we re-analyzed the phylogeographic study of each lineage separately and included more E gene sequences retrieved from the same BLAST search performed in the previous analysis. The ML and BI analysis steps were the same as those described previously in Methods. In BI analysis,we only reduced the number of runs to 200,000,000 generations since this number was sufficient to reach good ESS values for each parameter sampled (ESS >200). The best fit for the nucleotide substitution model for both lineage samples was TN93+G. *Clocklikeness* was determined using the ML trees as input. The correlation coefficient and substitution rate were 0.8729 and 6.68 95E-4 in lineage 1 samples and 0.8858and 6.8374E-4 in lineage 2 samples, respectively. The high correlation coefficient in both analyses demonstrates that both datasets fit to a strict molecular clock model. Phylogenetic trees generated from each lineage dataset using the ML method (data not shown) presented a topology similar to that of the respective phylogenetic trees generated using the BI method (Figure 4 and 5).

The addition of more samples in our phylogeographic analysis resulted in new values on the estimative of the time to MRCA and also revealed other evolutionary events relevant for describing the introduction and transmission of DENV-2 in Brazil. Discrete phylogeographic analysis detailing the lineage 1 (Figure 4) showed that the introduction of this lineage in Brazil most likely occurred in Rio de Janeiro in 1987 (95% HPD  = 1985–1988; modal state posterior probability  = 70.77%) (Figure 4, Node 1), two years earlier than estimated in the previous phylogeographic analysis (Figure 2, Node 1). The virus then spread to other Brazilian states, including Piauí, and it has been introduced to other countries in South America, such as Paraguay and Peru. Another distinctive aspect in this tree lies in the basal position of the lineage. We observe an early divergence between the isolates from Brazilian Southeast region (Rio de Janeiro and Espírito Santo states) and Northeast region (Pará, Ceará and Rio Grande do Norte states. The evolutionary history of PI-111 could not be clearly traced using phylogenetic analysis with the currently available DENV-2 samples in the GenBank database. However, the tree demonstrates that PI-111 is more related to a sample from Paraguay (Paraguay/EU045313/2005), and we can estimate that the introduction of DENV-2 in Piauí occurred in approximately 2003 (95% HPD  = 2001–2004) (Figure 4, Node 2), four years later than estimated in the previous phylogeographic analysis (Figure 2, Node 2).

In the phylogenetic tree detailing the evolutionary history of lineage 2 (Figure 5), Brazilian samples shared the same MRCA with samples from Jamaica (GQ199892/2007, JF804034/2008 and AB545873/2007) and St. Vincent (JF968043/2010), which are inferred to have existed since 2003 (95% HPD  = 2001–2005) (Figure 5, Node 1). In this tree, Brazilian isolates can be divided into two subgroups. One subgroup is represented by a unique early diverging sample from northern Brazil (BR/Northern/GQ199890/2008), referred as lineage 2b. The other group consists of all other Brazilian samples that have clustered together with isolates previously delineated as lineage 2, now referred as lineage 2a. In the later, isolates from Piauí (PI-55, PI-58, PI-59, PI-62, PI-74, PI-82 and PI-83) was also in basal position of the lineage. The MRCA of the samples from lineage 2a is estimated to have existed since 2004 (95% HPD  = 2003–2005), as has been inferred in the previous analysis (Figure 2, Node 3), with a high modal state posterior probability of being from Piauí (93.39%) (Figure 5, Node 2). This suggests a dissemination route for this lineage in which DENV-2 passed through the state of Piauí before causing later outbreaks in other Brazilian states (Rio de Janeiro, São Paulo, Espírito Santo and Bahia) and also in other countries of South America (Peru, Paraguay and Bolivia).

## Discussion

Various genomic regions of DENV have been used for molecular phylogenetic analyses [11]–[12], [40]–[43], and the E gene appears to have epidemiologically relevant sequence information [11], [41]–[42]. In this work, we have reconstructed the phylogeographic history of DENV-2 in Brazil, and for the first time, we have included samples from the state of Piauí collected during the 2006/2007 outbreak. Our study shows that all DENV-2 isolates obtained from this outbreak were grouped within the American-Asian genotype together with viruses from South and Central America, including those from the Caribbean islands and the southeastern states of Brazil (São Paulo and Rio de Janeiro) [44], [20]–[22]. This genotype has been established as the major lineage of DENV-2 in Central and South America and has been described as a genotype with a high epidemiological impact due to its ability to spread and its potential to cause DHF [11].

The isolates from Piauí were separated into two different lineages (lineages 1 and 2), indicating temporal co-circulation of two genetically distinct DENV-2 lineages in this state. The co-circulation of more than one DENV-2 lineage has been described in Central and South America [44]–[45]. Among the six aa changes observed in the E protein between samples from lineages 1 and 2 three were non-conservative, while those at positions 129, 203 and 380 were conservative (Figure 3). Most aa substitutions found in our DENV-2 samples were observed in American-Asian genotype isolates and provide evidence of the Caribbean origin of Piauí samples, as do the genotype/lineage markers of the E gene from other Brazilian samples [46], [45], [20]. Notably, residue 131 in the E gene is located within a pH-dependent hinge region at the interface between domains I and II of the envelope protein. Mutations in this region may affect the pH threshold of fusion and the process of conformational change [47]. Only sample 74 had a serine (H), polar neutral, at position 429, while the other samples had phenylalanine (F), which is neutral and hydrophobic. This change increased the hydrophobicity of the protein. Because this change is located in domain III of the E protein, alterations in the binding of the virus to cell receptors may result.

The phylogeographic analysis presented here revealed that the American-Asian genotype DENV-2 was introduced into Brazil more than once since 1990, which agrees with previous studies [21]–[22]. The introduction of lineage 1 in Brazil was estimated to have occurred in 1987 (Figure 4, Node 2) in Rio de Janeiro after which this lineage disseminated to other Brazilian states, including Piauí. Lineage 1 was detected predominantly from 1990–2003. However, one isolate from Piauí that belongs to lineage 1 (PI-111/2006) was detected in 2006. Our detailed analysis reveals that more recent samples of this lineage were detected in other countries, such as Paraguay (Figure 4), which leads us to hypothesize that a new introduction event occurred in Brazil in 2003 (Figure 4, Node 2). It can also be speculated that this lineage was replaced by a more recent lineage of DENV-2 (lineage 2) in Piauí. The disproportion in the number of samples observed in each lineage (1 sample in lineage 1 and 7 samples in lineage 2) strengthens this assumption. Carrillo-Valenzo [48] also reported multiple introductions of viral lineages of various DENV serotypes in Mexico and frequent lineage replacement events.

A previous study suggested that lineage 2 could have been introduced into Brazil from the Caribbean islands via two parallel events: one in the southeast region in 2005 (Figure 5, lineage 2a) and another from the northern region (Figure 5, lineage 2b). This possibility was suggested because samples from both regions diverged early in the phylogeographic analysis [22]. Our results also demonstrate that these parallel events may have occurred. However, when we added sequences of isolates from the Piauí outbreak (2006/2007) into the phylogeographic analysis, those samples clustered together with samples from the southeast region (Figure 2 and lineage 2a). Moreover, the samples from Piauí were positioned at the base of the branch between the introduction of this lineage in Brazil and the first epidemic described in the southeast region, which occurred in the state of Rio de Janeiro during the years 2007–2008 [20]. Thus, there is a high probability that DENV-2 entered through the state of Piauí, in 2006/2007 suggesting a new route of introduction and dissemination of this lineage in Brazil. This hypothesis was also suggested in the phylogeographic analysis of lineage 1 (Figure 4), since samples from the northeast region are situated in basal position of the lineage.

We suggested that from Piauí, this virus disseminated to other Brazilian states, such as Rio de Janeiro and São Paulo, and some countries in South America, such as Peru and Bolivia. Moreover, our analysis demonstrates that outbreaks that occurred in other states, such as Rio de Janeiro and São Paulo (southeast region),originated from Piauí, supporting the idea that the introduction of this DENV-2 lineage in Brazil occurred before other outbreaks were described in Brazil (2007, 2008 and 2010). The state of Piauí is located in the northeast region and is near the Caribbean region, which could be considered the point of introduction and dissemination of a new DENV in Brazil. The close geographic proximity of the state of Piauí to the Caribbean region may explain the considerable influx of DENV-2 lineages across the northeastern Brazilian border. On the other hand, lineage 1 was most likely introduced through the southeastern Brazilian region [20]–[21].

In conclusion, our study demonstrates that all the DENV-2 isolated in Piauí during the 2006/2007 outbreak clustered within the American-Asian genotype and confirmed for the first time the occurrence of spatiotemporal co-circulation of two distinct lineages of DENV-2. Both lineages (lineage 1 and 2) identified in this work were most likely independently introduced into this state. The Caribbean islands are the main source of DENV-2 viruses in Brazil, and northeastern Brazil appears to be an important route of introduction and dissemination of this virus in the country. These findings can help us to further understand the complex phylogeographic history of dengue viruses and their evolution in dengue endemic regions.

## Supporting Information

Figure S1 **Average temporal distribution of precipitation and suspected or confirmed dengue cases.**
(EPS)

Figure S2 **Evolutionary relationships of DENV-2 based on the envelope nucleotide sequence.** ML tree of 72 DENV-2 E genes including all five genotypes: American-Asian, American, Asian I, Asian II and Cosmopolitan. The taxon label is presented in the following format: country/GenBank accession number/year. Samples from Brazil are identified as follows: BR-city-state/GenBank accession number/year of isolation (in bold blue letters). Isolates from Piauí/Brazil are indicated with a red arrow. Phylogenetic tree reconstruction using ML methods was performed using PhyML with the Tamura-Nei model with gamma correction (TN93+G) and a bootstrap test with 100 replicates. Bootstrap supporting values greater than 50 are shown at the nodes.
(EPS)

Table S1 **GenBank accession numbers for DENV-2 sequences used in phylogenetic, phylogeographic and coalescent analyses, by country and year of isolation.**
(XLSX)

Table S2 **Pairwise distance based on E gene nucleotide sequence among DENV-2 isolates from Piauí state and others viruses isolated worldwide.** All values are in percentage of identity. Lower and higher values of identity are represented by red and green color respectively. Intermediate values are represented by yellow.
(XLS)
