# Correction: Dengue Virus 2 American-Asian Genotype Identified during the 2006/2007 Outbreak in Piauí, Brazil Reveals a Caribbean Route of Introduction and Dissemination of Dengue Virus in Brazil

**DOI:** 10.1371/journal.pone.0113777

**Published:** 2014-11-17

**Authors:** The PLOS ONE Staff

Figure S1 is incorrect. Please view the correct Figure S1 below.

The seventh sentence of the second paragraph of the Discussion section is also incorrect. The correct sentence is: Only sample 74 had a serine (S), polar neutral, at position 429, while the other samples had phenylalanine (F), which is neutral and hydrophobic.

## Supporting Information

Figure S1 **Average temporal distribution of precipitation and suspected or confirmed dengue cases.**
(TIF)
