# Supplementary figures and images for: Correction: Dengue Virus 2 American-Asian Genotype Identified during the 2006/2007 Outbreak in Piauí, Brazil Reveals a Caribbean Route of Introduction and Dissemination of Dengue Virus in Brazil
Source: PLoS One. 2014 Nov 17;9(11):e113777. doi: 10.1371/journal.pone.0113777 (PMC4234657; doi:10.1371/journal.pone.0113777)

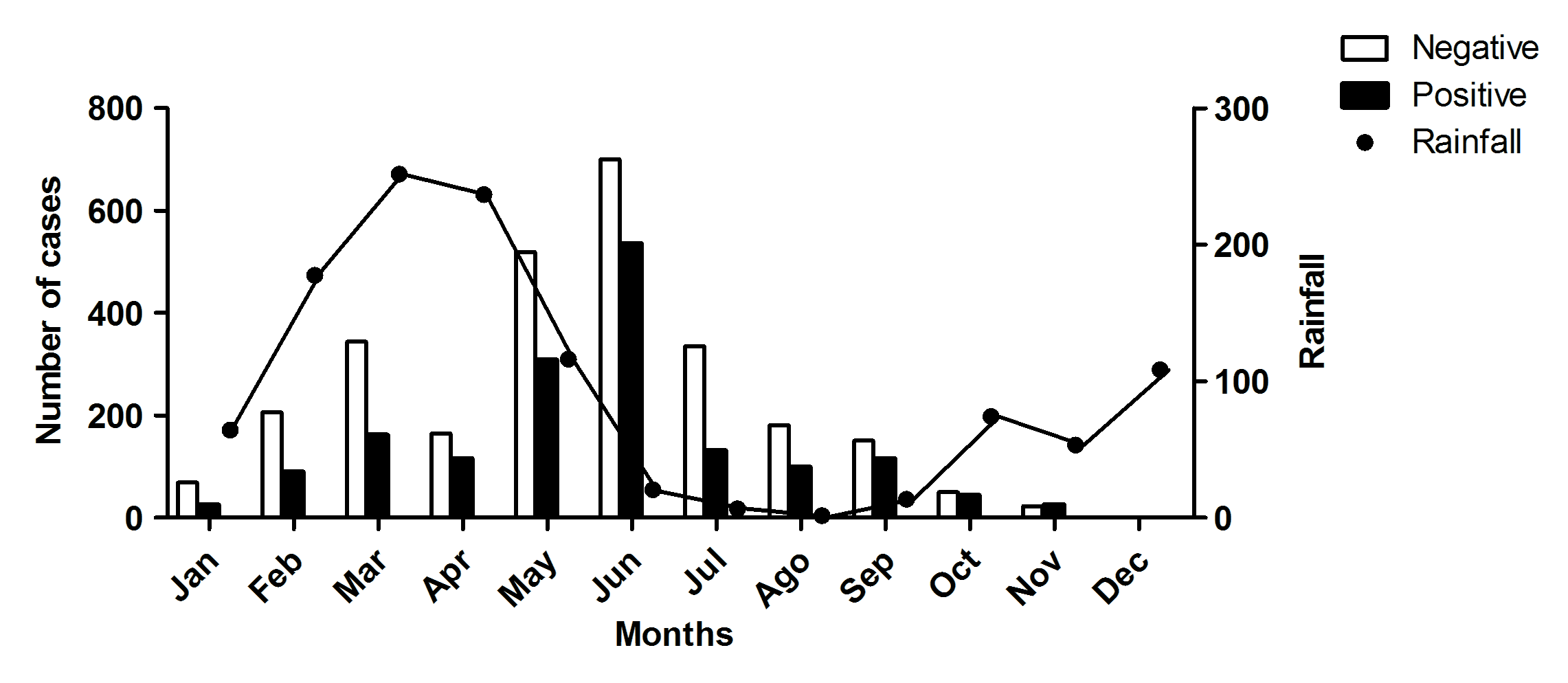

Supplement: Figure S1 — Average temporal distribution of precipitation and suspected or confirmed dengue cases. (TIF) [file pone.0113777.s001.tif]
